# Indirect Impact of Pandemic on the Diagnosis of New Primary Melanoma: A Retrospective, Multicenter Study

**DOI:** 10.3390/jcm14062017

**Published:** 2025-03-16

**Authors:** Luca Nespoli, Lorenzo Borgognoni, Virginia Caliendo, Dario Piazzalunga, Piero Rossi, Marco Clementi, Stefano Guadagni, Corrado Caracò, Serena Sestini, Maria Gabriella Valente, Franco Picciotto, Cosimo Di Raimondo, Davide Ferrari, Irene Tucceri Cimini, Amy Giarrizzo, Salvatore Asero, Matteo Mascherini, Franco De Cian, Francesco Russano, Paolo Del Fiore, Francesco Cavallin, Sara Coppola, Elisabetta Pennacchioli, Pietro Gallina, Marco Rastrelli

**Affiliations:** 1 General and Emergency Surgery, School of Medicine and Surgery, Milano-Bicocca University, Fondazione IRCCS San Gerardo dei Tintori, Via Pergolesi 33, 20900 Monza, Italy; luca.nespoli@unimib.it; 2 Plastic and Reconstructive Surgery Unit, Regional Melanoma Referral Center and Melanoma & Skin Cancer Unit, Santa Maria Annunziata Hospital, 50012 Florence, Italy; lorenzo.borgognoni@uslcentro.toscana.it (L.B.); serena.sestini@uslcentro.toscana.it (S.S.); 3 Department of Surgery, Dermatologic Surgery Section, Azienda Ospedaliera Universitaria (AOU) Città della Salute e della Scienza, 10126 Turin, Italy; virginiacaliendo@gmail.com (V.C.); franco.picciotto@gmail.com (F.P.); 4 Unit of Surgery, Papa Giovanni XXIII Hospital, 24100 Bergamo, Italy; dpiazzalunga@asst-pg23.it (D.P.); ferraridav93@gmail.com (D.F.); 5 Dipartimento Di Scienze Chirurgiche, Università di Roma Tor Vergata, Policlinico Tor Vergata Viale Oxford 81, 00133 Rome, Italy; piero.rossi.box@icloud.com; 6 General Surgical Unit, Department of Biotechnological and Applied Clinical Sciences, University of L’Aquila, Via Vetoio, 67100, L’Aquila, Italy; marco.clementi@univaq.it (M.C.); stefano.guadagni@univaq.it (S.G.); irene.tuccericimini@graduate.univaq.it (I.T.C.); 7 Division of Surgery of Melanoma and Skin Cancer, Istituto Nazionale Tumori ‘Fondazione Pascale’ IRCCS, 80131 Naples, Italy; corrado.caraco@istitutotumori.na.it; 8 Unità di Anatomia Patologica, Fondazione IRCCS San Gerardo dei Tintori, 20900 Monza, Italy; mg.valente@hsgerardo.org; 9 Dermatology Unit, Fondazione Policlinico Tor Vergata, 00133 Rome, Italy; cosimodiraimondo@gmail.com; 10 Soft Tissue U.O. Surgical Oncology-Soft Tissue Tumors, Dipartimento di Oncologia, Azienda Ospedaliera di Rilievo Nazionale e di Alta Specializzazione Garibaldi Catania, 95123 Catania, Italy; amyg@hotmail.it (A.G.); s.asero@iol.it (S.A.); 11 Department of Surgical Sciences and Integrated Diagnostic DISC, University of Genoa, 16100 Genoa, Italy; matteo.mascherini@hsanmartino.it (M.M.); decian@unige.it (F.D.C.); 12 Department of Surgery, IRCCS Ospedale Policlinico San Martino, 16132 Genoa, Italy; 13 Soft-Tissue, Peritoneum and Melanoma Surgical Oncology Unit, Veneto Institute of Oncology IOV-IRCCS, 35128 Padova, Italy; francesco.russano@iov.veneto.it (F.R.); marco.rastrelli@unipd.it (M.R.); 14 Independent Statistician, 36020 Solagna, Italy; 15 Division of Melanoma, Soft Tissue Sarcomas and Rare Tumors, European Institute of Oncology, IRCCS, 20141 Milan, Italy; sara.coppola@ieo.it (S.C.); elisabetta.pennacchioli@ieo.it (E.P.); 16 Directorate General, Veneto Institute of Oncology IOV-IRCCS, 35128 Padua, Italy; pietro.gallina@iov.veneto.it; 17 Department of Surgery, Oncology and Gastroenterology (DISCOG), University of Padua, 35122 Padova, Italy

**Keywords:** melanoma, impact of pandemic, multicenter study

## Abstract

**Background/Objectives**: The indirect impact of the pandemic on the diagnosis and treatment of new primary melanoma has been carefully evaluated in recent years. The aim of the present study was to investigate if the indirect impact of the pandemic in Italy could be detectable also in the second year of the pandemic, as suggested by the characteristics of melanoma at diagnosis. **Methods**: Retrospective analysis of 1640 diagnoses of cutaneous melanoma in pre-pandemic period and 1292 diagnoses in the pandemic period from 10 centers (from 1 March 2019 to 28 February 2022). **Results**: Our findings confirmed an indirect impact of the pandemic on characteristics of incident melanoma, also in the second year of the pandemic in Italy (Breslow thickness *p* < 0.0001, tumor stage *p* = 0.002, ulceration *p* = 0.04, SNLB *p* = 0.03), without statistically significant differences between centers. A statistically significant reduction in the time interval from diagnosis to surgical treatment was observed, but only in centers that had to modify their case mix to address the needs of treating COVID-19 patients (*p* = 0.0002). **Conclusions**: Our study confirmed the indirect impact of the pandemic on melanoma characteristics at the diagnosis in the second year of the pandemic in Italy. We also found no differences in melanoma characteristics between hospitals with different organization. Diagnostic delays may be related to a delayed access of the patient to the entire diagnostic pathway, and therefore, especially in the case of a pandemic, policies to support early diagnosis are crucial.

## 1. Introduction

Melanoma is a severe and potentially fatal skin cancer, which is particularly sensitive to delays in diagnosis and treatment. The prognosis for melanoma patients is significantly influenced by early diagnosis and prompt treatment. More advanced T and N stages in melanoma correlate with worse disease-free survival and a higher risk of recurrence. While patients with melanoma less than 1 mm in depth and localized to the skin have a 93–97% five-year survival rate, those with late-stage distant metastatic melanoma face drastically lower survival rates of 10–20% over five years, depending on the metastasis location [1]. One of the key prognostic factors at the time of diagnosis is the melanoma’s growth phase, whether vertical or radial: the association between the Breslow thickness of the primary melanoma and prognosis is well established [2,3,4]. Other prognostic variables with a significant impact include the presence of ulceration (which is incorporated into the current staging system) and the mitotic rate (measured as mitoses per mm^2^), given its prognostic relevance across all thickness categories [5]. On 11 March 2020, the World Health Organization announced that the COVID-19 outbreak had reached the level of a global pandemic [6]. Several international studies showed that patients have presented with higher-stage melanoma since the start of the pandemic, suggesting delayed diagnosis and more advanced disease at presentation, such as increased Breslow thickness, ulceration rate and tumor staging [7,8,9,10]. Several Italian studies have shown similar results after the lockdown [11,12,13].

Indeed, in response to pandemic, many countries around the world tried to limit the spread of the virus and reduce the risk of infection, both reorganizing healthcare services and imposing mobility restrictions to the whole population. These interventions seemed to have a positive impact on COVID-19 diffusion [14] but, on the other hand, significantly disrupted healthcare services worldwide, leading to reductions in the number of patient visits, diagnostic testing, imaging, and therapeutic care [15].

Despite a real-life retrospective observational study showing that, while reduced in number, dermatological surgery remained operative in Italy to provide oncological procedures [16], the lockdown significantly impacted outpatient visits during the first peak of the pandemic, with many visits being delayed or missed [17]. The pandemic peak might have impacted on the number of patients requesting a dermatology outpatient visit [18]. Such disruptions can be accounted for in the indirect impact of the pandemic, in contrast to the direct impact, including sickness, mortality, and long-term health issues [19].

Studies from various regions reported major reductions in melanoma diagnoses and a decrease in skin biopsy volumes during the pandemic [20,21,22]. Recent data indicated that approximately 12,700 new cases of melanoma are diagnosed annually in Italy [11], but there was a decline in new melanoma diagnoses during the first phase of the COVID-19 pandemic (lockdown), which was approximately 20% lower than pre-pandemic levels [23]. The hypothesis about the association of fear of visiting hospitals and the reduction in the number of melanoma diagnoses after the lockdown could also be supported by the larger reduction in Northern and Central Italy, where the incidence of COVID-19 was higher.

Although from the second year of the pandemic onward, healthcare system activities gradually returned to normal, partly due to vaccination campaigns, no multicenter studies in Italy have investigated, in a high number of cases, whether the characteristics of melanoma at the time of the diagnosis observed during the first year of the pandemic were still present. The aim of the present study was to investigate if higher-stage melanoma at the time of the diagnoses could be detectable also in the second year of the pandemic in Italy in comparison with the pre-pandemic period. In addition, the study explored if the organization characteristics during the pandemic in different skin cancer centers in Italy could have an impact on the characteristics of melanoma at the time of diagnosis. In fact, some Italian hospitals were designated as “COVID-19 centres” during the pandemic, acting as treatment hubs with dedicated beds for COVID-19 patients. In such hospitals, most efforts were devoted to COVID-19 patients and surgical management was restricted to critical interventions such as oncological procedures. On the other hand, many Italian hospitals had no COVID-19 dedicated beds and did not change their surgical case mix during the pandemic. We hypothesized that skin cancer centres located in COVID-19 centres might have been affected by the change in the surgical case mix during the pandemic.

## 2. Materials and Methods

### 2.1. Study Design

This is a retrospective, observational, multicenter study evaluating the indirect impact of the COVID-19 pandemic on the presentation of melanoma patients in nine Italian melanoma centers. The study was conducted in accordance with Resolution no. 9/2016 of the Italian Data Protection Authority (Authorization no. 9/2016—General authorization for the processing of personal data carried out for scientific research purposes), which established the admissibility of processing personal data for medical, biomedical, and epidemiological research, that data relating to the health status of individuals may be used in aggregate form in scientific studies, and that the acquisition of informed consent for data processing is not necessary if it is not possible to contact the data subjects in order to provide information due to reasons of organizational impossibility attributable to the circumstances of conducting the study, in particular in relation to the period of time that has elapsed since the data relating to the data subjects were originally collected.

All analyses were conducted on secondary, aggregated, and anonymized data (retrospective reviews of registers), resulting from eliminating any possibility of individual identification using an anonymization tool.

### 2.2. Patients

Data for all patients who were diagnosed and/or treated for primary cutaneous melanoma in four COVID-19-free centers and five COVID-19 centers between 1 March 2021 and 1 March 2022 (pandemic period) were collected and compared to the same period of 2019–2020 (pre-pandemic period) and included in the study. The main inclusion criteria were histologically confirmed diagnosis of cutaneous melanoma, age ≥ 18 years, and clinical stage I–III.

### 2.3. Study Periods

In this study, the pre-pandemic period included all consecutive primary histological diagnoses of cutaneous melanoma from 1 March 2019 to 28 February 2020, while the pandemic period under evaluation (second year) included all consecutive primary histological diagnoses of cutaneous melanoma from 1 March 2021 to 28 February 2022. Two different kinds of Melanoma Centre Units were involved in the study. Skin cancer centers located in hospitals dedicated to oncological diseases, without COVID-19 dedicated beds, which substantially had no changes in their case mix during the pandemic, in our analysis were defined as “COVID-19-free centers”. On the other hand, skin cancer centers located in hospitals not exclusively dedicated to oncological diseases, with COVID-19 dedicated beds, where the surgical case mix changed during the pandemic, were defined as “COVID-19 centers” in our analysis.

### 2.4. Outcome Measures

Breslow thickness, tumor stage, intratumoral and peritumoral tumor-infiltrating lymphocytes (TILs), mitosis, and ulceration were included as clinically relevant tumor characteristics at the diagnosis. The time interval from the diagnosis to the surgery was included as an indicator of clinical pathway timing. The proportion of patients who underwent sentinel lymph node biopsy (SLNB) and those with positive nodes and the number and the largest size of the positive nodes (tumor burden) were included as indicators of disease progression at diagnosis All data were extracted from the hospital charts and collected in an anonymized database for the analysis.

### 2.5. Statistical Analysis

Numerical data were summarized as mean and standard deviation (SD), while categorical data were summarized as absolute and relative frequency (percentage). The indirect impact of the COVID-19 pandemic was assessed using linear regression models and logistic regression models, including the period (pandemic vs. pre-pandemic), the center classification (COVID-19 vs. COVID-19-free), and the interaction term (period × center). The effect sizes were calculated as mean difference (MD) or odds ratio (OR) with 95% cluster-robust confidence interval (CI). The effect size for the period estimated the change from the pre-pandemic to the pandemic period. The effect size for the center classification estimated the baseline difference between COVID-19 and COVID-19-free centers. The effect size for the interaction term estimated the different change over time between COVID-19 and COVID-19-free centers. The relationship between patient’s age and time interval from diagnosis to surgery according to pre-pandemic and pandemic periods was investigated by adding patient’s age and the interaction term “period × age” in the model described before. The effect size for this interaction estimated the different change over time of the correlation between patient’s age and time interval from diagnosis to surgery. A secondary analysis of the indirect impact of the pandemic stratified by sex was also conducted with linear regression models and logistic regression models including the period (pandemic vs. pre-pandemic), the sex (males vs. females) and the interaction term (period × sex). The effect size for the interaction term estimated the different change over time between males and females. All tests were two-sided and a *p*-value less than 0.05 was considered statistically significant. The statistical analysis was carried out with R (version 4.3, 2023) (R Foundation for Statistical Computing, Vienna, Austria) [24].

## 3. Results

The analysis included 1640 diagnoses of cutaneous melanoma in the pre-pandemic period and 1292 diagnoses in the pandemic period. Overall, the study sample included 1640 men and 1292 women aged 18–98 years. Patient’s age was distributed in 18–29 years (*n* = 75, 3%), 30–39 years (*n* = 203, 7%), 40–49 years (*n* = 447, 15%), 50–59 years (*n* = 669, 23%), 60–69 years (*n* = 599, 20%), 70–79 years (*n* = 586, 20%), 80–89 years (*n* = 321, 11%), and 90–99 years (*n* = 27, 1%). Patient’s age was unspecified in five patients. The diagnoses were performed in four COVID-19-free centers (1143 diagnoses before the pandemic and 827 diagnoses in the pandemic period) and five COVID-19 centers (497 diagnoses before the pandemic and 465 diagnoses in the pandemic period).

### 3.1. Interval from Diagnosis to Surgery

In COVID-19-free centers, the mean interval from diagnosis to surgery was 67.9 days (SD 50.6) in the pre-pandemic period and 67.5 days (SD 35.8) in the pandemic period (Table 1). In COVID-19 centers, the mean interval was 68.9 days (SD 112.2) in the pre-pandemic period and 56.3 days (SD 36.3) in the pandemic period (Table 1). The interval changed from the pre-pandemic period to the pandemic period among centers (Table 2), with a decreasing interval in COVID-19 centers compared to COVID-19-free centers (MD −12.1 days, 95% CI −18.4 to −5.7 days; *p* = 0.0002). Of note, the data did not suggest any differences in the indirect impact of the pandemic on the correlation between time interval from diagnosis to surgery and patient’s age (interaction term “period × age”: MD 0.0 days, 95% CI −0.2 to 0.2 days; *p* = 0.73).

### 3.2. Breslow Thickness

Summary statistics for Breslow thickness in the pre-pandemic period and the pandemic period are reported for COVID-19-free and COVID-19 centers in Table 1. Breslow thickness increased from the pre-pandemic period to the pandemic period (MD 0.3 mm, 95% CI 0.2 to 0.5; *p* < 0.0001), but the change was not statistically different between COVID-19-free and COVID-19 centers (*p* = 0.17) (Table 2).

### 3.3. Tumor Stage

Summary statistics for the tumor stage in the pre-pandemic period and the pandemic period are reported for COVID-19-free and COVID-19 centers in Table 1. Diagnoses of stage II–III increased from the pre-pandemic period to the pandemic period (OR 1.45, 95% CI 1.14 to 1.85; *p* = 0.002), but the change was not statistically different between COVID-19-free and COVID-19 centers (*p* = 0.27) (Table 2). Similarly, diagnoses of N+ stage increased from the pre-pandemic period to the pandemic period (OR 1.45, 95% CI 1.13 to 1.85; *p* = 0.003), but the change was not statistically different between COVID-19-free and COVID-19 centers (*p* = 0.16) (Table 2).

### 3.4. Tumor-Infiltrating Lymphocytes (TILs)

The information was not available in two COVID-19-free centers which were excluded from the analysis of this outcome measure. Summary statistics for the TILs in the pre-pandemic period and the pandemic period are reported for COVID-19-free and COVID-19 centers in Table 1.

The presence of intratumoral TILs (TILs Brisk) did not significantly change from the pre-pandemic period to the pandemic period (*p* = 0.14) and there were no statistically significant different changes between COVID-19-free and COVID-19 centers (*p* = 0.42) (Table 2).

Similarly, the presence of peritumoral TILs (TILs non-BRISK) did not significantly change from the pre-pandemic period to the pandemic period (*p* = 0.97) and there were no statistically significant different changes between COVID-19-free and COVID-19 centers (*p* = 0.15) (Table 2).

### 3.5. Mitosis

Summary statistics for mitosis in the pre-pandemic period and the pandemic period are reported for COVID-19-free and COVID-19 centers in Table 1. Mitosis increased from the pre-pandemic period to the pandemic period (MD 0.6 unit/mm^2^, 95% CI 0.4 to 0.9; *p* < 0.0001), but the change was not statistically different between COVID-19-free and COVID-19 centers (*p* = 0.91) (Table 2).

### 3.6. Ulceration

Summary statistics for the ulceration in the pre-pandemic period and the pandemic period are reported for COVID-19-free and COVID-19 centers in Table 1. The presence of ulceration increased from the pre-pandemic period to the pandemic period (OR 1.22, 95% CI 1.01 to 1.48; *p* = 0.04), but the change was not statistically different between COVID-19-free and COVID-19 centers (*p* = 0.51) (Table 2).

### 3.7. SLNB

Summary statistics for SLNB in the pre-pandemic period and the pandemic period are reported for COVID-19-free and COVID-19 centers in Table 1. The proportion of patients who underwent SLNB increased from the pre-pandemic period to the pandemic period (OR 1.33, 95% CI 1.02 to 1.74; *p* = 0.03), but the change was not statistically different between COVID-19-free and COVID-19 centers (*p* = 0.12) (Table 2).

### 3.8. SLNB Positivity

In patients who underwent SLNB, summary statistics for SLNB positivity in the pre-pandemic period and the pandemic period are reported for COVID-19-free and COVID-19 centers in Table 1. The proportion of patients with positive SLNB among those who underwent SLNB increased from the pre-pandemic period to the pandemic period (OR 1.26, 95% CI 1.01 to 1.57; *p* = 0.04), but the change was not statistically different between COVID-19-free and COVID-19 centers (*p* = 0.80) (Table 2).

### 3.9. Number of Positive Nodes

In patients with positive SLNB, summary statistics for the number of positive nodes in the pre-pandemic period and the pandemic period are reported for COVID-19-free and COVID-19 centers in Table 1. The number of positive nodes differently changed from the pre-pandemic period to the pandemic period among centers (Table 2), with an increasing number of positive nodes in COVID-19 centers compared to COVID-19-free centers (MD 0.4, 95% CI 0.1 to 0.8; *p* = 0.002).

### 3.10. Largest Size of Positive Nodes (Tumor Burden)

In patients with positive SLNB, summary statistics for the tumor burden in the pre-pandemic period and the pandemic period are reported for COVID-19-free and COVID-19 centers in Table 1. The variation of the tumor burden from the pre-pandemic period to the pandemic period was not statistically different between COVID-19-free and COVID-19 centers (MD 1.6 mm, 95% CI −0.2 to 3.4 mm; *p* = 0.08) (Table 2).

### 3.11. Secondary Analysis of the Indirect Impact of the Pandemic Stratified by Sex

Summary statistics for the outcome measures in the pre-pandemic and pandemic periods are reported for males and females in Table 3. This secondary analysis did not suggest any differences in the indirect impact of the pandemic between males and females (as shown in the “interaction term” column of Table 4).

## 4. Discussion

### 4.1. COVID-19 Pandemic in Italy

On 11 March 2020, the World Health Organization declared a coronavirus outbreak as a global pandemic. Most countries worldwide implemented actions to face the spread of the disease, including health service reorganization to make resources available to manage COVID-19 patients and mobility limitations to reduce the risk of infection. In Italy, the first locally infected cases of COVID-19 were identified on 20 and 21 February 2020, respectively, in Codogno and Schiavonia (Northern Italy). On March 9, rigid limitations on population mobility were applied in part of the country and very soon were extended to the entire country to control the spread of infections. On 10 March 2020, the Italian Health Ministry gave specific indications for oncologic care [25], recommending local health authorities to organize specific and dedicated pathways and areas for oncologic care, separated from other patients. Development of specific strategies to guarantee a prompt diagnosis and therapy for oncological patients with SARS-CoV-2 infection was recommended. At the same time, patients were invited to delay follow-up visits when possible and in agreement with oncologists to reduce the risk of infection. On 16 March 2020, the Health Ministry published guidelines [26] to reorganize elective outpatient visits and surgical procedures. To protect patients and healthcare professionals from in-hospital virus transmission, non-oncological elective hospitalizations and delayable outpatient activities, including screening, were delayed. Only from 1 June 2020 did a gradual reopening of services begin with recovery of both inpatient and outpatient activities. Further delays were evident with the second pandemic peak at the end of the year. Indeed, new limitations were introduced during the second wave of the COVID-19 pandemic, between October 2020 and February 2021.

Therefore, based on this chronological progression, the pre-pandemic period of our study included the diagnoses up to 28 February 2020. As previously mentioned, our study focused on the mid-term effects of the COVID-19 pandemic on melanoma diagnosis and treatment, specifically during its second year. This period was marked by the widespread rollout of vaccination campaigns and a gradual return to normality for health services in Italy.

### 4.2. The Impact of the COVID-19 Pandemic on Skin Cancer Screening and Diagnosis

Many studies investigated the impact of the pandemic on cancer diagnosis and management. Despite some heterogeneity, many papers reported a possible delay in diagnosis for several different forms of cancer [27,28,29].

Some studies reported the same effect also for skin cancer diagnosis [30,31], with a higher number of patients presenting with advanced disease stages. A Turkish study found a negative correlation between the number of COVID-19 diagnoses in the country and the number of patients requesting a dermatology outpatient visit [16]. A recent meta-analysis on European studies suggested an association between delayed diagnosis of cutaneous melanoma related and increased Breslow thickness, ulceration rate, and tumor staging [7]. Our data confirmed such findings as Breslow thickness, ulceration, stage II–III, lymph node involvement at diagnosis, and patients with positive SLNB increased from the pre-pandemic to the pandemic period according to data previously published in other studies [7,8,9,10].

### 4.3. Diagnosis Delay and Organization During the Pandemic

We suppose that these characteristics may mirror a possible diagnostic delay related to the difficulty in accessing the health services due to multiple factors such as social distancing, movement limitation, fear of infection, and health services reorganization. Regarding the outcomes of interest, our data did not show a different indirect impact of the pandemic in males and females. Similarly, our data did not suggest a shift to a younger or older cohort in the delayed surgical procedures due to the pandemic. The most common clinical pathway for diagnosing melanoma, indeed, is represented by initial evaluation by a general practitioner, followed by referral to a dermatologist and/or a surgeon for a definitive diagnosis. We believe that the previous factors might have impaired the access to the first dermatological visit during the first two waves of the pandemic, thus resulting in delay in the first step of the clinical pathway and more advanced disease at the diagnosis. Moreover, the reduction in the number of diagnoses between pre-pandemic and pandemic periods, especially in “not COVID-19-free” hospitals, may also mirror patient difficulty in accessing diagnostic and treatment pathways. Nonetheless, we cannot exclude that pre-existing conditions might have influenced the diagnostic delay, but unfortunately such data were not available for further analyses. Despite some differences in incidence across regions, the pandemic had an impact on hospital organization throughout the whole country. Hence, our study also compared the indirect impact of the pandemic in skin cancer centers with differences in organization. Our data suggested that the interval from diagnosis to surgery decreased from the pre-pandemic period to the pandemic period in skin cancer centers located in hospitals that were not dedicated to oncological diseases, where the surgical case mix changed during the pandemic (“COVID-19 centers”), On the other hand, the interval from diagnosis to surgery did not change in skin cancer centers located in hospitals dedicated to oncological diseases which had not substantially changed their case mix during the pandemic (“COVID-19-free centers”). The reorganizations inside COVID-19 hospitals led to a different case mix in the operating rooms, which likely influenced the decrease in the waiting time for oncological diseases like melanoma. The follow-up of these data in the next years could confirm such a hypothesis.

### 4.4. Strengths and Implications for Future Pandemics

The strengths of our study include the multicenter design, which allowed us to obtain significantly high numbers, and the fact that it is based on “real-life” data.

This study has also some limitations that should be considered. First, the generalizability of the findings should be limited to similar healthcare settings. Second, the retrospective data collection might have limited the data quality. Although our data can be extrapolated to healthcare systems such as the Italian one, findings related to Breslow thickness, ulceration, stage II–III disease, and lymph node involvement at diagnosis also apply to significantly different healthcare models.

Our results confirmed this effect during the second year of the pandemic, and it is reasonable to assume that similar trends may be observed in other settings as well. A prospective follow-up of these data could help determine the time required to restore melanoma diagnostic characteristics to pre-pandemic baseline levels. These findings underscore the need for organizational strategies to ensure that the initial step in the melanoma diagnostic and treatment pathway—dermatologic assessment—is not compromised. The adoption of emerging technologies, such as telemedicine, artificial intelligence-assisted diagnostics, or other innovative solutions, could serve as valuable tools to mitigate diagnostic delays in malignant melanoma during future pandemics, thereby preventing long-term repercussions on patient outcomes [32,33,34,35].

Finally, our multicentric study confirmed the indirect impact of the pandemic on melanoma characteristics at the diagnosis in the second year of the pandemic, extending some observations that have been described in the first phase of the pandemic. We also found no differences in melanoma characteristics and staging at presentation between COVID-19-free and COVID-19 centers, suggesting that policies to preserve access to dermatological visits for moles are necessary and have to be preserved in both COVID-19-free and COVID-19 Centers in case of pandemics, also using teledermatoscopy. The diagnosis process was not influenced by being a dedicated COVID-19 center or not; instead, the reduction in waiting times for the COVID-19 centers is due to the decision to exclude benign surgical pathologies in favor of malignant pathologies. Delays in melanoma diagnosis during the pandemic could have significant long-term consequences for melanoma patients: many patients who missed early screenings may present with thicker, more aggressive melanomas (higher Breslow index, ulceration, and mitotic rate); early-stage melanoma is highly treatable, but delays can allow metastasis, leading to reduced survival and increased mortality, and late diagnoses can lead to increased emotional distress, anxiety, and reduced quality of life. Addressing these consequences requires systemic healthcare reforms, strategies to implement teledermatology, awareness campaigns, and prioritization of routine cancer screenings to prevent similar backlogs in future health crises.

## 5. Conclusions

Our findings suggested that the pandemic had an indirect impact on the diagnosis of more aggressive melanomas in Italy, while the reorganization within COVID-19 hospitals reduced the time interval from diagnosis to surgery. When considering the public health implications of the pandemic, future interventions should prevent the increase in late diagnoses by ensuring continued access to dermatology care, classifying skin cancer screenings as essential medical services to avoid interruptions, and implementing dedicated pathways for melanoma diagnosis during pandemics to prioritize high-risk patients. In addition, implementing and standardizing teledermatology may address healthcare system vulnerability by integrating teledermatology consultations into routine care and providing training for primary care physicians to conduct initial skin assessments when specialist visits are limited.

## Figures and Tables

**Table 1:** Summary of the outcome measures in the pre-pandemic period (from March 2019 to February 2020) and the pandemic period (from March 2021 to February 2022) stratified by COVID-19-free centers and COVID-19 centers.

Outcome Measure	COVID-19-Free Centers		COVID-19 Centers	
	Pre-Pandemic Period (*n* = 1143)	Pandemic Period (*n* = 827)	Pre-Pandemic Period (*n* = 497)	Pandemic Period (*n* = 465)
Time interval from diagnosis to surgery, days: mean (SD)	67.9 (50.6)	67.5 (35.8)	68.9 (112.2)	56.3 (36.3)
Breslow thickness, mm: mean (SD)	1.5 (2.7)	1.9 (2.7)	1.7 (2.7)	1.9 (2.6)
Stage II–III: n/N (%)	249/1120 (22.2%)	223/812 (27.5%)	73/459 (15.9%)	114/444 (25.7%)
Stage N+: n/N (%)	89/1131 (7.9%)	81/814 (10.0%)	30/462 (6.5%)	51/448 (11.4%)
TIL Brisk: n/N (%) ^a^	216/538 (40.1%)	136/279 (48.7%)	249/381 (65.3%)	264/388 (68.0%)
TIL non-Brisk: n/N (%) ^a^	231/481 (48.0%)	147/280 (52.5%)	278/360 (60.4%)	190/334 (56.9%)
Mitosis, unit/mm^2^: mean (SD)	2.3 (4.8)	3.0 (5.3)	1.9 (2.8)	2.4 (3.7)
Ulceration: n/N (%)	169/1067 (15.8%)	143/746 (19.2%)	65/373 (17.4%)	70/364 (19.2%)
Patients who underwent SLNB: n/N (%)	454/1131 (40.1%)	354/811 (43.6%)	138/463 (29.8%)	195/447 (43.6%)
Patients with positive SLNB: n/N (%)	87/454 (19.2%)	78/353 (22.1%)	29/135 (21.5%)	51/193 (26.4%)
Number of positive nodes among patients with positive SLNB: mean (SD)	1.4 (0.7)	1.2 (0.5)	1.1 (0.3)	1.4 (0.8)
Tumor burden, mm: mean (SD) ^b^	2.6 (2.9)	2.4 (2.9)	2.1 (2.1)	3.6 (4.0)

SD: standard deviation. SLNB: sentinel lymph node biopsy. TIL: tumor-infiltrating lymphocyte. The information was not available in ^a^ 2 and ^b^ 1 COVID-19-free centers that were excluded from the analysis of the outcome measure.

**Table 2:** Results from the regression models: the effect size for the period estimated the change from the pre-pandemic to the pandemic period; the effect size for the center classification estimated the baseline difference between COVID-19 and COVID-19-free centers; the effect size for the interaction term (period × center) estimated the different change over time between COVID-19 and COVID-19-free centers.

Outcome Measure	Effect Size	Period: Pandemic vs. Pre-Pandemic	Center: COVID-19 vs. COVID-19-Free	Interaction Term (Period × Center)
Time interval from diagnosis to surgery, days	MD (95% cluster-robust CI)	−0.5 (−1.7 to 0.6)	0.9 (−13.2 to 14.9)	−12.1 (−18.4 to −5.7) *
Breslow thickness, mm	MD (95% cluster-robust CI)	0.3 (0.2 to 0.5) *	0.0 (−0.4 to 0.4)	−0.2 (−0.4 to 0.1)
Stage II–III	OR (95% cluster-robust CI)	1.45 (1.14 to 1.85) *	0.78 (0.42 to 1.47)	1.38 (0.78 to 2.43)
Stage N+	OR (95% cluster-robust CI)	1.45 (1.13 to 1.85) *	0.99 (0.53 to 1.84)	1.43 (0.87 to 2.34)
TIL Brisk	OR (95% cluster-robust CI)	1.27 (0.93 to 1.74)	2.55 (0.37 to 17.76)	0.80 (0.46 to 11.39)
TIL non-Brisk	OR (95% cluster-robust CI)	1.01 (0.52 to 1.99)	1.16 (0.18 to 7.46)	0.46 (0.16 to 1.32)
Mitosis, unit/mm^2^	MD (95% cluster-robust CI)	0.6 (0.4 to 0.9) *	−0.6 (−1.1 to −0.1)	0.0 (−0.5 to 0.4)
Ulceration ^a^	OR (95% cluster-robust CI)	1.22 (1.01 to 1.48) *	1.06 (0.81 to 1.40)	0.89 (0.64 to 1.24)
Patients who underwent SLNB: n/N (%)	OR (95% cluster-robust CI)	1.33 (1.02 to 1.74) *	0.79 (0.27 to 3.32)	1.58 (0.88 to 2.82)
Patients with positive SLNB: n/N (%)	OR (95% cluster-robust CI)	1.26 (1.01 to 1.57) *	1.22 (0.92 to 1.79)	1.10 (0.54 to 2.23)
Number of positive nodes among patients with positive SLNB	MD (95% cluster-robust CI)	−0.2 (−0.4 to 0.1)	−0.2 (−0.4 to −0.1) *	0.4 (0.1 to 0.8) *
Tumor burden, mm	MD (95% cluster-robust CI)	−0.2 (−0.6 to 0.3)	−0.5 (−1.6 to 0.6)	1.6 (−0.2 to 3.4)

CI: confidence interval. MD: mean difference. SLNB: sentinel lymph node biopsy. TIL: tumor-infiltrating lymphocyte. OR: odds ratio. * *p* < 0.05. Pre-pandemic period: from March 2019 to February 2020; pandemic period: from March 2021 to February 2022. The information was not available in ^a^ 2 COVID-19-free centers that were excluded from the analysis of the outcome measure.

**Table 3:** Summary of the outcome measures in the pre-pandemic period (from March 2019 to February 2020) and the pandemic period (from March 2021 to February 2022) stratified by sex.

Outcome Measure	Females		Males	
	Pre-Pandemic Period (*n* = 727)	Pandemic Period (*n* = 565)	Pre-Pandemic Period (*n* = 913)	Pandemic Period (*n* = 727)
Time interval from diagnosis to surgery, days: mean (SD)	70.5 (95.6)	60.7 (32.0)	66.4 (45.2)	65.2 (39.5)
Breslow thickness, mm: mean (SD)	1.3 (2.1)	1.8 (2.6)	1.6 (3.1)	2.0 (2.6)
Stage II–III: n/N (%)	120/707 (17.0%)	133/549 (24.2%)	202/972 (23.2%)	204/707 (28.8%)
Stage N+: n/N (%)	45/712 (6.3%)	52/551 (9.4%)	74/881 (8.4%)	80/711 (11.2%)
TIL Brisk: n/N (%) ^a^	206/396 (52.0%)	196/317 (61.8%)	259/523 (49.5%)	204/350 (58.3%)
TIL non Brisk: n/N (%) ^a^	198/399 (49.6%)	134/276 (48.6%)	311/542 (57.4%)	203/338 (60.0%)
Mitosis, unit/mm^2^: mean (SD)	1.9 (4.5)	2.5 (4.4)	2.4 (4.2	3.0 (5.2)
Ulceration: n/N (%)	82/631 (13.0%)	74/747 (15.6%)	152/809 (18.8%)	139/636 (21.9%)
Patients who underwent SLNB: n/N (%)	245/712 (34.4%)	225/550 (40.9%)	347/882 (39.3%)	324/708 (45.8%)
Patients with positive SLNB: n/N (%)	44/244 (18.0%)	52/225 (23.1%)	72/345 (20.9%)	77/321 (24.0%)
Number of positive nodes among patients with positive SLNB: mean (SD)	1.3 (0.7)	1.3 (0.7)	1.3 (0.7)	1.3 (0.6)
Tumor burden, mm: mean (SD)	2.6 (2.5)	3.0 (3.5)	2.3 (2.8)	2.9 (3.5)

SD: standard deviation. SLNB: sentinel lymph node biopsy. TIL: tumor-infiltrating lymphocyte. The information was not available in ^a^ 2 COVID-19-free centers that were excluded from the analysis of the outcome measure.

**Table 4:** Results from the regression models: the effect size for the period estimated the change from the pre-pandemic to the pandemic period; the effect size for the sex estimated the baseline difference between males and females; the effect size for the interaction term (period × sex) estimated the different change over time between males and females.

Outcome Measure	Effect Size	Period: Pandemic vs. Pre-Pandemic	Sex: Males vs. Females	Interaction Term (Period × Sex)
Time interval from diagnosis to surgery, days	MD (95% cluster-robust CI)	−9.7 (−20.4 to 1.0)	−4.1 (−14.8 to 6.5)	8.6 (−3.1 to −20.3)
Breslow thickness, mm	MD (95% cluster-robust CI)	0.3 (0.2 to 0.5) *	0.3 (0.1 to 0.6) *	−0.1 (−0.6 to 0.3)
Stage II-III	OR (95% cluster-robust CI)	1.43 (1.12 to 1.82) *	1.13 (1.13 to 1.66) *	0.86 (0.61 to 1.22)
Stage N+	OR (95% cluster-robust CI)	1.44 (1.14 to 1.83) *	1.28 (0.95 to 1.74)	0.89 (0.50 to 1.60)
TIL Brisk	OR (95% cluster-robust CI)	1.47 (0.86 to 2.52)	1.33 (1.16 to 1.52) *	0.86 (0.60 to 1.25)
TIL non-Brisk	OR (95% cluster-robust CI)	1.04 (0.54 to 1.98)	1.45 (1.21 to 1.74) *	1.16 (0.69 to 1.96)
Mitosis, unit/mm^2^	MD (95% cluster-robust CI)	0.6 (0.3 to 0.9) *	0.5 (0.1 to 0.9)	0.0 (−0.6 to 0.6)
Ulceration	OR (95% cluster-robust CI)	1.22 (1.01 to 1.47) *	1.53 (1.34 to 1.76) *	0.98 (0.60 to 1.59)
Patients who underwent SLNB: n/N (%)	OR (95% cluster-robust CI)	1.31 (1.00 to 1.71) *	1.23 (1.03 to 1.41) *	0.99 (0.77 to 1.27)
Patients with positive SLNB: n/N (%)	OR (95% cluster-robust CI)	1.26 (1.01 to 1.57) *	1.20 (0.84 to 1.49)	0.88 (0.48 to 1.60)
Number of positive nodes among patients with positive SLNB	MD (95% cluster-robust CI)	0.0 (−0.2 to 0.2)	0.0 (−0.2 to 0.2)	0.0 (−0.3 to 0.3)
Largest size of positive nodes, mm	MD (95% cluster-robust CI)	0.4 (−0.9 to 1.7)	−0.3 (−1.4 to 0.8)	0.1 (−1.1 to 1.4)

CI: confidence interval. MD: mean difference. OR: odds ratio. SLNB: sentinel lymph node biopsy. TIL: tumor-infiltrating lymphocyte. * *p* < 0.05. Pre-pandemic period: from March 2019 to February 2020; pandemic period: from March 2021 to February 2022.

## Data Availability

The datasets presented in this study can be found in online repositories. The names of the repository/repositories and accession number(s) can be found here: https://zenodo.org/records/14004256 (accessed on 15 February 2025).
